# Mapping the Global Surge in Postoperative Sleep Research From 2014 to 2024: Bibliometric Analysis

**DOI:** 10.2196/86086

**Published:** 2026-03-26

**Authors:** Xin Wei, Lijuan Fu, Wencai Jiang, Xianjie Zhang, Rui Zhou

**Affiliations:** 1 Department of Anesthesiology, Deyang People's Hospital, Deyang, China; 2 Department of Anesthesiology and Perioperative Medicine, Shanghai Fourth People's Hospital, No 1279, Sanmen Road, Hongkou District, Shanghai, China, 86 18108812438

**Keywords:** bibliometric analysis, postoperative sleep, recovery quality, postoperative pain, enhanced recovery after surgery

## Abstract

**Background:**

Postoperative sleep is closely associated with recovery among patients undergoing surgery.

**Objective:**

This study aimed to analyze the research status and developmental trends in postoperative sleep between 2014 and 2024.

**Methods:**

Publications were retrieved from the Web of Science Core Collection. Microsoft Excel and VOSviewer were used to analyze the papers in terms of publication trends, countries, institutions, authors, journals, and keywords.

**Results:**

A total of 964 papers were obtained for the bibliometric analysis. The number of publications on this topic has increased gradually over the last 10 years. Zhu Junchao was the most prolific author in the field, and Chung Frances had the most citations. China had the most publications, followed by the United States. Scientific institutions in China, such as China Medical University and Capital Medical University, have led the way in terms of publication numbers. A total of 40 journals have published at least 5 papers. *BMC Anesthesiology*, with 19 publications, ranked first in publication count. The papers published in the *British Journal of Anaesthesia*, *Journal of Clinical Anesthesia*, *Anesthesia and Analgesia*, *Journal of Pain*, and *Journal of Sleep Medicine* had higher citation counts on average. The high-frequency keywords were “sleep quality,” “postoperative pain,” “quality of life,” and “surgery,” while “lung cancer,” “enhanced recovery after surgery,” “breast cancer,” and “dexamethasone” emerged as new topics in this area.

**Conclusions:**

There has emerged a large body of literature on postoperative sleep over the past 10 years. Authors and organizations from China are leading contributors, followed by those from the United States. Anesthesiology is a critical discipline in this field. Postoperative pain is closely related to postoperative sleep and has become a major research focus. Recent studies have mainly focused on lung cancer and breast cancer surgeries. Enhanced recovery after surgery has become an emerging keyword.

## Introduction

Postoperative sleep shows a close relationship with the quality of recovery, hormone secretion, metabolism, cognitive function, and autonomic nervous activity [1]. Maintenance of normal sleep after surgery is closely related to postoperative rehabilitation [2]. However, patients undergoing surgery may experience a degree of impact on their sleep quality [3]. Anesthesia has the potential to disrupt the sleep-wake timing system [4,5], as most general anesthetics are either N-methyl-D-aspartate receptor antagonists or γ-aminobutyric acid agonists, and activation of these receptors affects clock gene expression [6]. Glucocorticoids, synthetic analogs of endogenous cortisol, are sometimes administered during surgery and have strong effects on the molecular clock [7]. In addition, the grade and duration of surgery have been demonstrated to determine the degree of postoperative sleep disorders [8,9]. Thus, normal sleep after surgery may be difficult to maintain because of the multifaceted effects of surgery and anesthesia on sleep during the early postoperative period.

Postoperative sleep disorders have received increasing attention from researchers [10]. More than 1900 publications were recorded in the Web of Science (WoS) Core Collection from 2014 to 2023. Despite the increasing number of studies, a comprehensive and systematic overview of the global research status, developmental trends, and research hot spots in postoperative sleep remains lacking. Previous studies have mainly focused on specific clinical issues such as the correlation between sleep quality and postoperative pain or the effect of a single intervention on sleep improvement. However, there is a lack of macrolevel analyses summarizing the research landscape, identifying collaborative networks, and predicting future research directions in this field.

Bibliometric analysis is a commonly used method for evaluating research trends [11]. To date, no bibliometric study has been conducted to systematically analyze the global literature on postoperative sleep over the past decade. Therefore, this study aimed to fill this gap by performing a bibliometric analysis of publications on postoperative sleep from 2014 to 2024. The specific research goals are as follows: (1) to characterize publication trends and identify key contributing countries, institutions, authors, and journals in this field; (2) to identify the core research hot spots and emerging topics; and (3) to provide a reference for researchers to grasp the global research status and formulate future research directions. The findings of this study will not only help academic researchers identify research gaps but also provide evidence-based insights for clinicians to optimize perioperative management strategies and improve patient recovery outcomes.

## Methods

### Ethical Considerations

This study did not include human or animal participants; therefore, ethics approval was not required for this study. For this type of study, formal consent was also not required.

### Identification of Papers on Postoperative Sleep

This study was a bibliometric analysis based on an accessible database. The WoS Core Collection database was systematically retrieved on May 6, 2025, to search for publications on postoperative sleep. The search terms were as follows: TS= (postoperative sleep disorder) OR TS= (postoperative sleep disturbance) OR TS= (postoperative insomnia) OR TS= (postoperative agrypnia) OR TS= (postoperative sleeplessness) OR TS= (postoperative hyposomnia) OR TS= (postoperative sleep quality). The time span was from 2014 to 2024.

Studies were excluded if they were non-English publications, publications on preoperative sleep disorders, non–surgery-related publications, publication types other than research papers and review papers, retracted publications, or publications on animal experiments. Two investigators independently searched and screened the records. The review process included two parts: (1) title and abstract screening (the 2 investigators independently reviewed the title and abstract of each retrieved publication) and (2) full-text verification (for publications with ambiguous descriptions in the titles and abstracts, the full texts were further retrieved and evaluated). If discrepancies arose, a third reviewer was consulted to resolve them through discussion or arbitration.

### Data Collection, Cleaning, and Analysis

Eligible publications were extracted. The extracted items included the title, authors, country or region, journal, affiliation, keywords, and publication year. The files were downloaded in “.txt” and “.xls” formats and then imported into VOSviewer (version 1.6.20) and Microsoft Excel (version 2021). All data extraction procedures were performed on the same day to avoid bias caused by database updates. In addition, the 2 investigators manually collected the specializations of the first authors.

Subsequently, we created thesaurus files to merge synonyms for countries and keywords. For example, “sleep disorders” and “insomnia” were replaced by “sleep disturbance.” Thus, VOSviewer could automatically recognize and merge the duplicate terms. Quantitative analyses of the data, such as annual publication numbers and citation counts, were performed by Microsoft Excel.

VOSviewer was used to build a collaborative network of authors, countries, institutions, and journals to study collaborative linkages and the intensity of collaboration among different nodes, as well as to perform a clustering analysis of the frequently cocited keywords. For country, institution, and author collaboration networks, analysis type was coauthorship; the minimum number of papers per country, institution, and author were 5, 5, and 3, respectively; the normalization method was association strength; and the display style (meaning of the color) per country, institution, and author was by average publishing year, by average publishing years, and by cooperation strength, respectively. For the journal collaboration network, the analysis type was cocitation, the minimum number of papers per journal was 5, the normalization method was association strength, and the display style was by average publishing year. For the keyword co-occurrence network, the analysis type was co-occurrence, the minimum number of occurrences of a keyword was 10, the normalization method was association strength, and the display style was by average publishing year.

## Results

### Overview of the Literature Related to Postoperative Sleep

**Figure 1.**
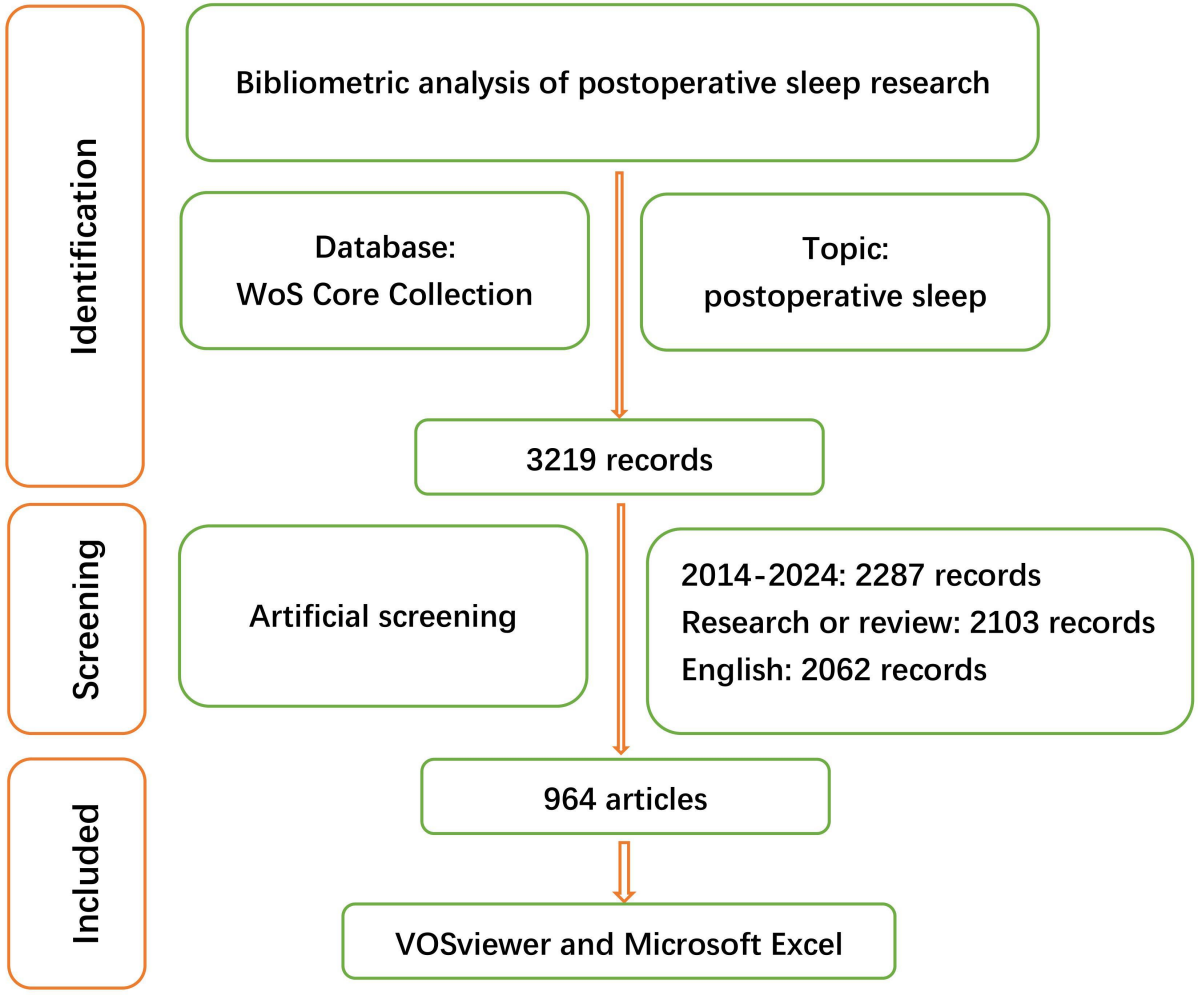
Flowchart demonstrating the selection process of this study. WoS: Web of Science.

An initial search indicated that there were 3219 publications that may be eligible in the WoS Core Collection database. Among these publications, 2287 (71.0%) were published between 2014 and 2024. After removing non-English publications and other paper types, 2062 (64.1%) studies were reviewed by reading the titles and abstracts, and 1098 (53.2%) studies were excluded according to the exclusion criteria, with 964 (46.8%) studies finally included in this analysis. The details of this selection process are shown in Figure 1.

### Annual Trend of Publication Quantity

**Figure 2.**
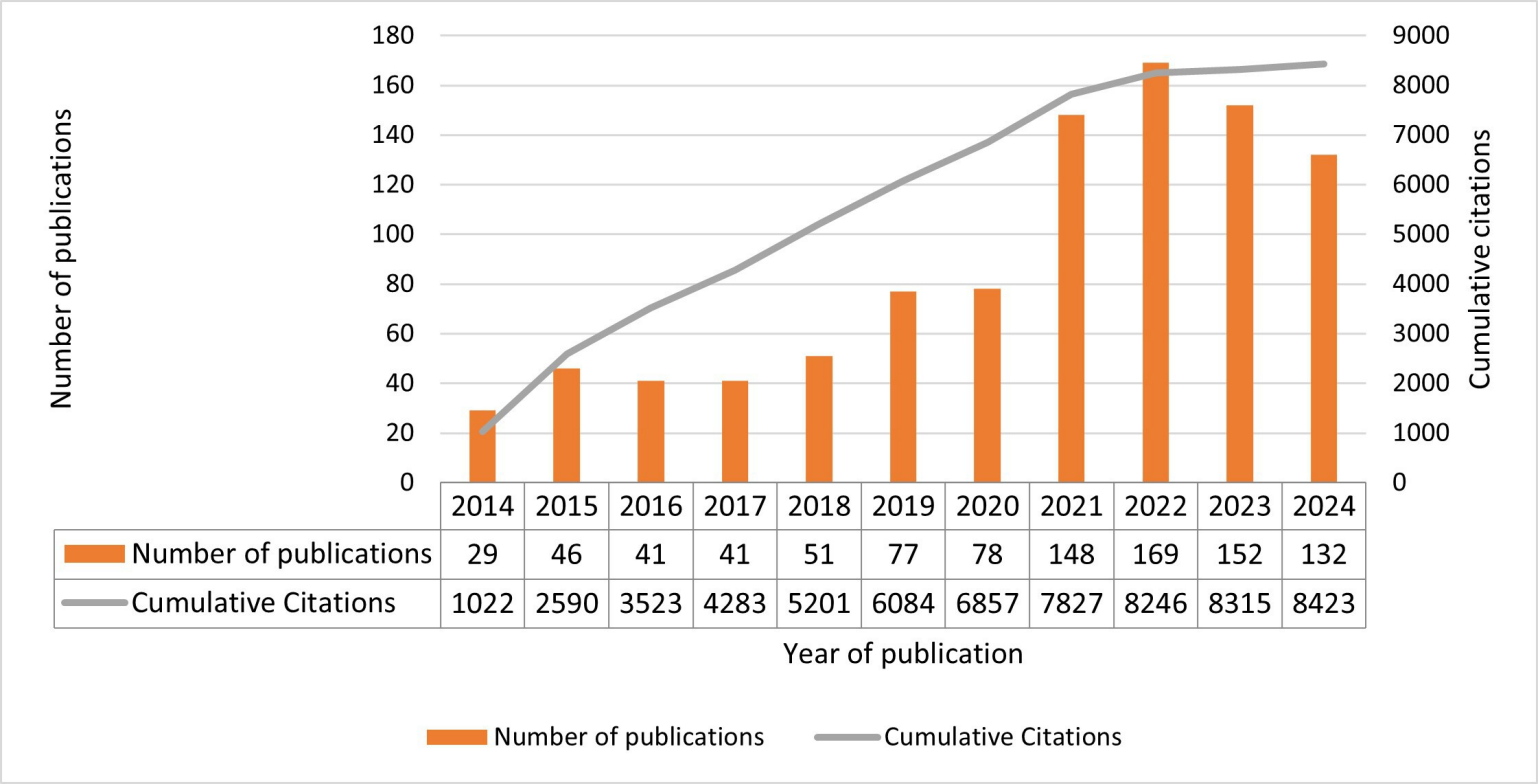
Annual number of publications and cumulative citations.

Postoperative sleep research has surged over the last decade. Figure 2 shows a substantial increase in annual publications, soaring from just 29 publications in 2014 to 169 publications in 2024. With a mean of 87.6 publications per year and a median of 77 publications per year, the field saw steady growth, punctuated by a significant surge in 2020. Research output has remained consistently high ever since, and these publications have already garnered an impressive 8423 citations.

### Contributions of Countries or Regions to Global Publications

In the last decade, 54 countries or regions contributed to the research on postoperative sleep. The top 10 countries or regions with the highest number of publications are listed in Table 1. China (n=402, 41.7%) made the highest contribution in terms of the number of publications, followed by the United States (n=201, 20.9%); Turkey (n=50, 5.2%); South Korea (n=48, 5.0%); Japan (n=35, 3.6%); Canada (n=26, 2.7%); Germany (n=25, 2.6%); Taiwan, China (n=23, 2.4%); Denmark (n=23, 2.4%); and Australia (n=19, 2.0%).

**Figure 3.**
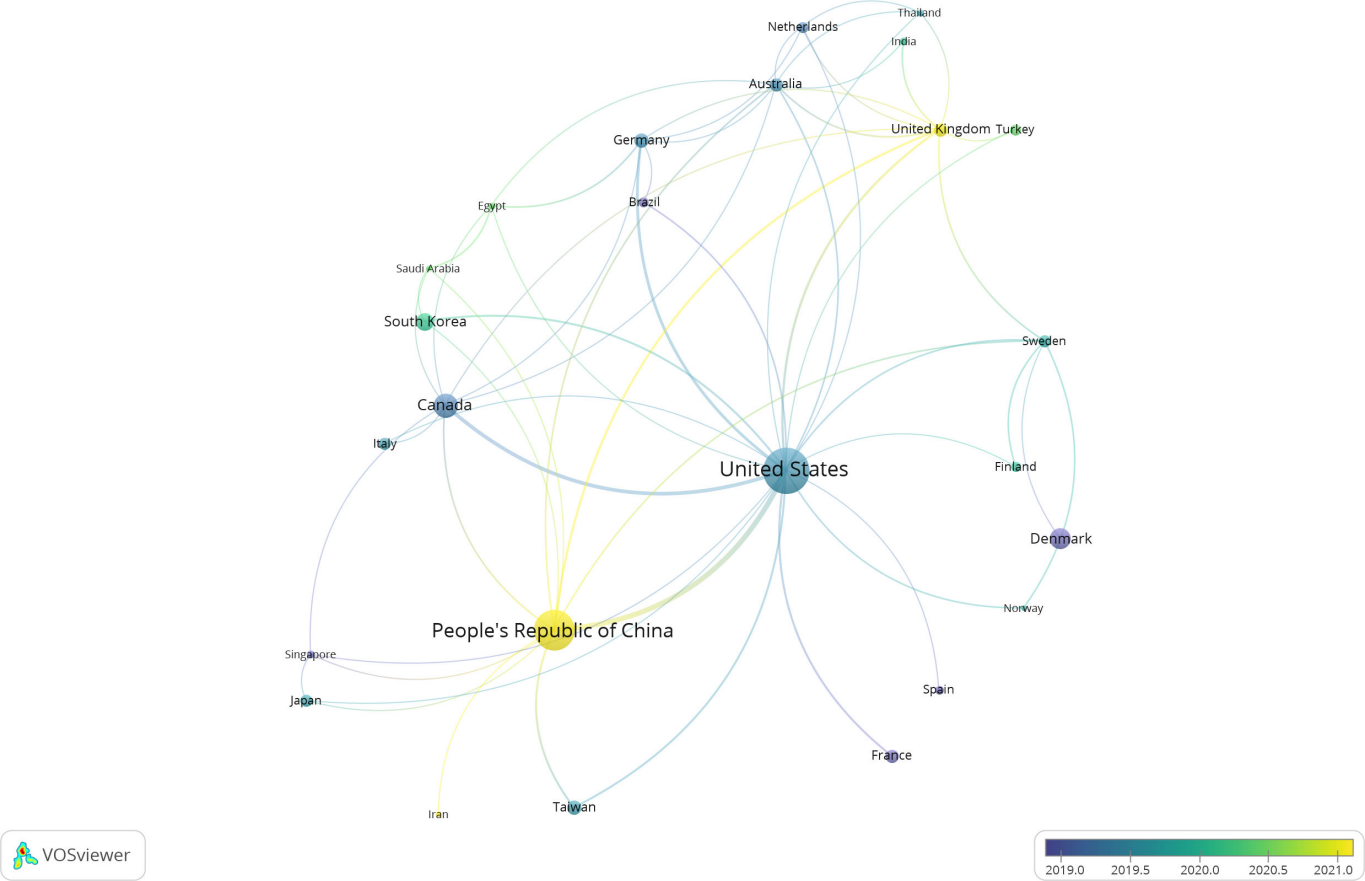
Links among the countries and regions. The nodes in the map have at least 5 papers.

The top 10 countries or regions in terms of citation count were the United States (n=2587); China (n=2144); Canada (n=699); Denmark (n=515); South Korea (n=389); Taiwan, China (n=269); Germany (n=257); Australia (n=230); the United Kingdom (n=214); and France (n=210; Table 2). The United States had the most extensive collaboration links with other countries or regions, and the collaboration between the United States and China was strongest (Figure 3). In addition, papers from China, the United Kingdom, and Iran had younger publication ages (Figure 3).

**Table 1.** The top 10 countries or regions in terms of publications.

Rank	Countries or regions	Publications, n
1	China	402
2	United States	201
3	Turkey	50
4	South Korea	48
5	Japan	35
6	Canada	26
7	Germany	25
8	Taiwan, China	23
9	Denmark	23
10	Australia	19

**Table 2.** The top 10 countries or regions in terms of citations.

Rank	Countries or regions	Citations, n
1	United States	2587
2	China	2144
3	Canada	699
4	Denmark	515
5	South Korea	389
6	Taiwan, China	269
7	Germany	257
8	Australia	230
9	United Kingdom	214
10	France	210

### Contributions of the Authors

**Figure 4.**
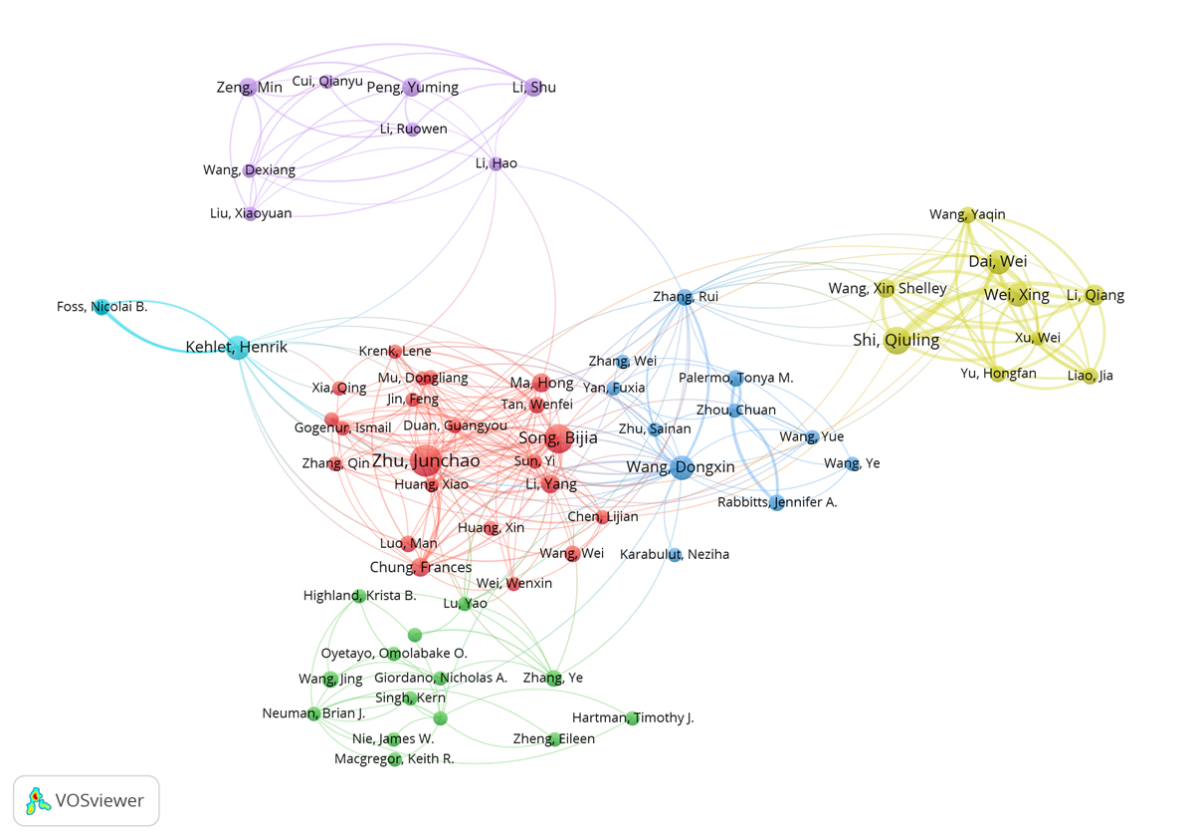
Citation analysis of the authors. The nodes in the map have at least 3 papers.

A total of 4677 authors published papers on postoperative sleep. Figure 4 shows 6 clusters, and the authors in each cluster had close citation relation. The top 10 authors in terms of publication number and citation count are listed in Tables3  4. In terms of publication number, Zhu Junchao ranked first, followed by Song Bijia, Shi Qiuling, and Kehlet Henrik. In terms of citation number, Chung Frances ranked first, followed by Kehlet Henrik, Mace Jess C, and Smith Timothy L. In addition, the first authors mainly specialized in anesthesiology, surgery, internal medicine, and nursing.

**Table 3.** The top 10 authors in terms of publications.

Rank	Authors	Publications, n
1	Zhu Junchao	17
2	Song Bijia	13
3	Shi Qiuling	11
4	Kehlet Henrik	10
5	Wang Dongxin	10
6	Dai Wei	9
7	Wei Xing	7
8	Mace Jess C	6
9	Smith Timothy L	6
10	Li Qiang	6

**Table 4.** The top 10 authors in terms of citations.

Rank	Authors	Citations, n
1	Chung Frances	248
2	Kehlet Henrik	227
3	Mace Jess C	219
4	Smith Timothy L	219
5	Shi Qiuling	161
6	Wang Dongxin	146
7	Zhu Junchao	122
8	Wang Xin Shelley	120
9	Palermo Tonya M	118
10	Rabbitts Jennifer A	118

### Contributions of the Affiliations

**Figure 5.**
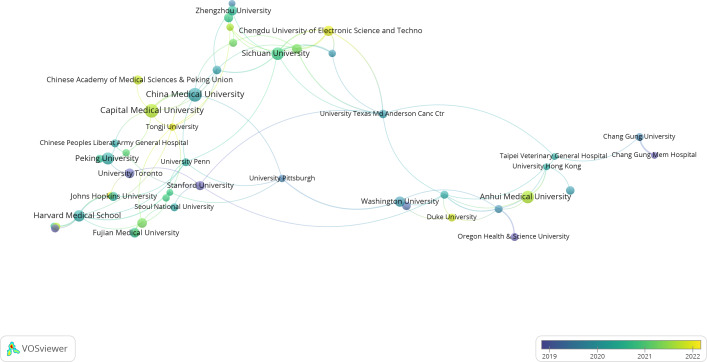
Citation relationships among institutions. The minimum number of papers per institution was set at 5.

A total of 1153 scientific institutions contributed to these papers. The top 10 affiliations in terms of publication number are listed in Table 5. China Medical University (n=24) ranked first, followed by Capital Medical University (n=23), Sichuan University (n=19), Anhui Medical University (n=18), the University of Copenhagen (n=18), and Peking University (n=18). In terms of citation number, the University of Copenhagen ranked first (n=488), followed by the University of Toronto (n=309), Peking University (n=232), and Oregon Health and Science University (n=224). Universities such as the University of Toronto and Stanford University were active in the 2010s, whereas universities such as Tongji University and Duke University have devoted themselves to this area more recently (Figure 5).

**Table 5.** The top 10 institutions with the most number of publications.

Rank	Institutions	Publications, n	Citations, n	Countries or regions
1	China Medical University	24	183	China
2	Capital Medical University	23	105	China
3	Sichuan University	19	148	China
4	Anhui Medical University	18	98	China
5	University of Copenhagen	18	488	Denmark
6	Peking University	18	232	China
7	Harvard Medical School	14	169	United States
8	University of Electronic Science and Technology of China	11	74	China
9	Washington University	11	161	United States
10	Chongqing Medical University	10	104	China
11	Shanghai Jiao Tong University	10	69	China
12	Zhengzhou University	10	97	China
13	University of Toronto	10	309	Canada
14	Fujian Medical University	10	121	China

### Distribution of the Journals

**Figure 6.**
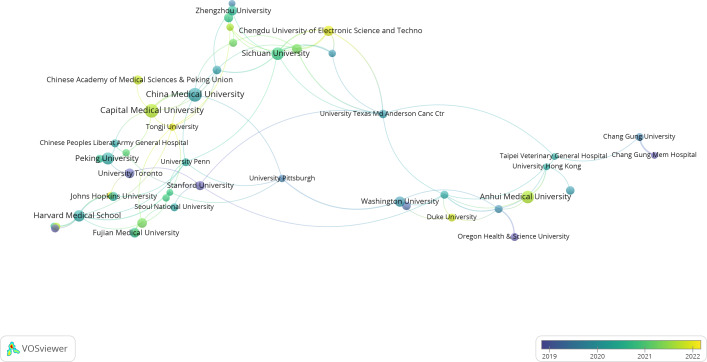
Citation relationships among journals. The minimum number of papers per journal was set at 5.

The papers on postoperative sleep in the last 10 years were documented by 392 journals indexed in the Scientific Citation Index. *BMC Anesthesiology,* with 19 publications, ranked first, followed by *Nature and Science of Sleep* (n=16) and *International Journal of Clinical and Experimental Medicine* (n=16). Papers published in the *British Journal of Anaesthesia*, *Journal of Clinical Anesthesia*, *Anesthesia and Analgesia*, *Journal of Pain*, and *Journal of Sleep Medicine* had higher citation counts on average (Figure 6).

### Analysis of Keywords

**Figure 7.**
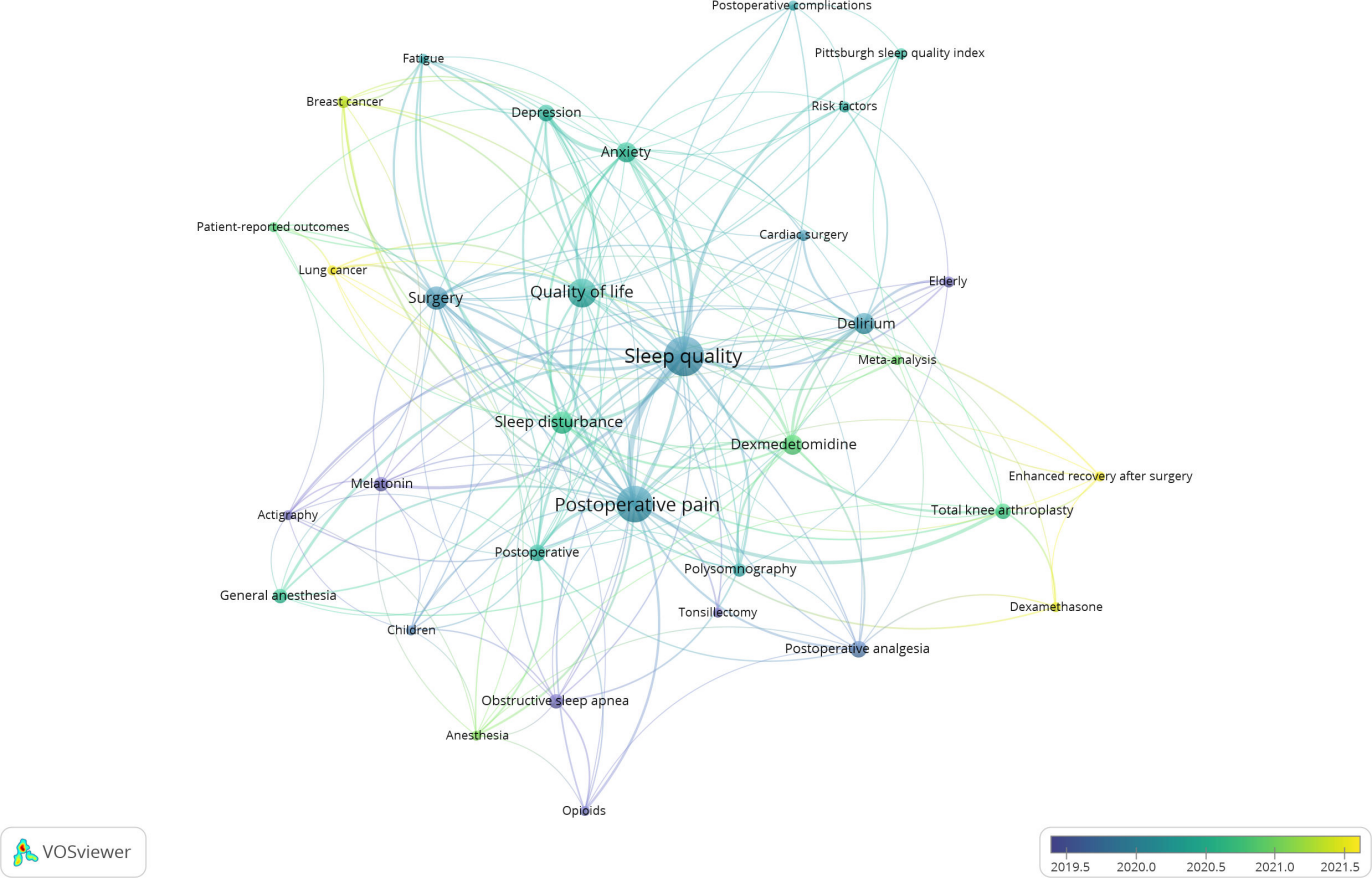
Co-occurrence analysis of author-provided keywords. The minimum occurrence threshold for inclusion in the map was set at 10.

The keyword co-occurrence analysis (Figure 7) revealed the primary research themes and focal points within the domain of postoperative sleep disturbances. The central and most prominent keywords were postoperative pain, sleep disturbance, and quality of life, indicating that these topics are core concerns driving research. Patient-reported outcomes also feature significantly, highlighting the importance of subjective patient experiences in this field.

Research methodologies are notably represented by specific assessment tools, including actigraphy (objective sleep measurement), polysomnography (the gold standard sleep study), and the Pittsburgh Sleep Quality Index (a common subjective sleep quality questionnaire). Key clinical contexts strongly associated with postoperative sleep issues included breast cancer, lung cancer, cardiac surgery, total knee arthroplasty, and tonsillectomy.

Pharmacological interventions such as dexmedetomidine and melatonin were also frequently identified. The concept of enhanced recovery after surgery (ERAS) appears frequently, suggesting that its protocols are being examined for their impact on preoperative sleep. Other significant keywords include “fatigue” and “delirium” (a serious postoperative complication potentially linked to sleep disturbance).

The temporal markers (2019.5 to 2021.5) indicate that this topic has received particular attention in recent years. “ERAS,” “lung cancer,” and “dexamethasone” emerge as more recent keywords in postoperative sleep research.

## Discussion

### Principal Findings

This bibliometric analysis focused on papers related to postoperative sleep over the past 10 years and provided an overview of publication trends, the distribution of publications, and hot research topics.

On the basis of the data collected in the WoS Core Collection, 964 papers on postoperative sleep were published during the past decade. With an increasing trend, the number of publications remained high. In total, 4677 authors from 1153 institutions across 54 countries or regions worldwide have published their work, and the number of researchers continues to grow. This trend and the distribution of publications suggest that postoperative sleep is an important focus in clinical practice and a prominent topic in the scientific literature, consistent with previous findings that postoperative sleep disorders can affect recovery [10,12,13].

China and the United States contributed the most to postoperative sleep research. They had the highest number of publications and citations and demonstrated the strongest international collaborations. However, fresh institutions in terms of average publication years mainly come from China, which indicates the expanding participation in this field. It is interesting that the top 10 authors and institutions in terms of publication number and citations were all from high-income countries or regions. This finding is not surprising but reasonable. A previous study was conducted to uncover the relationship between the development of anesthesia and gross domestic product from 2009 to 2018, and the results indicated a strong positive correlation [14].

Zhu Junchao is the most productive author in this field. The author has long been devoted to investigating the relationships between organ protection and cognitive impairment in the field of ischemia-reperfusion injury, postoperative outcomes, and sleep quality [15-17]. One of the related studies found that morning operations required a higher dose of anesthetics than evening operations and that the degree of postoperative sleep disorders was greater when the operation was performed in the evening than in the morning [17]. Meanwhile, the results prompted that patients with hyperpathia and somnipathy might benefit from operations performed in the morning [17,18]. Chung Frances, with 248 citations and only 5 publications, is the most cited author in the field of postoperative sleep research from 2014 to 2024. However, he and his research team have been engaged in postoperative sleep disorders for more than 10 years [13,19,20]. His paper published in *Anesthesiology* in 2014 revealed that the postoperative sleep structure was disturbed in both patients with obstructive sleep apnea and patients with nonobstructive sleep apnea, and they further analyzed disturbances in sleep structure [19]. His systematic review and meta-analysis also revealed the prevalence and risk factors of sleep disturbances in surgical patients [20]. These aforementioned authors are well respected as they greatly contribute to the prevention of postoperative sleep disturbance.

It was reported that the incidence of sleep disturbance was high (54%) during the postoperative period [21]. Determining the prevalence and risk factors related to postoperative sleep disturbance would be beneficial for risk stratification and perioperative care planning. On the basis of the keywords map, sleep quality was closely related to the postoperative quality of life, postoperative pain, delirium, anxiety, depression, dexmedetomidine use, and postoperative complications, while ERAS, lung cancer, breast cancer, and dexamethasone have received much attention recently. Postoperative pain is regarded as a primary risk factor for postoperative sleep disorders, and sleep quality can, in turn, aggravate pain [22-25].

The term “enhanced recovery after surgery” was coined by Henrik Kehlet, one of the most influential researchers in the field of postoperative sleep. Postoperative pain, a key component of ERAS protocols, and postoperative sleep have received considerable attention. Opioids, nonopioids, regional and neuraxial anesthesia, and multimodal analgesia based on the aforementioned techniques have been widely used in ERAS procedures, contributing to improved postoperative pain control [26,27]. Nevertheless, the incidence of postoperative sleep disorders is still high even though postoperative pain is well controlled [28,29]. Future studies are advised to build a systematic strategy for postoperative management.

To our knowledge, this is the first bibliometric analysis based on the WoS Core Collection to assess the studies on postoperative sleep over the past 10 years. This study included a large number of papers (n=964) on postoperative sleep to help identify the research status and hot spots. To improve the quality of the enrolled studies, 2 independent investigators conducted an artificial screening, and disagreements were properly settled. Moreover, duplicate terms were removed as far as possible by creating a thesaurus file.

The findings of this bibliometric analysis provide several practical implications for clinicians engaged in perioperative patient management. First, postoperative pain was identified as a core correlate of sleep disturbance in this study. Practitioners should integrate sleep quality assessment and interventions into ERAS pathways. They can combine multimodal analgesia with sleep-promoting strategies, such as melatonin administration or environmental optimization, to improve both pain control and sleep quality in surgical patients. Second, keyword analysis highlighted lung cancer and breast cancer surgeries as emerging research hot spots. Practitioners should pay special attention to sleep disorders in patients undergoing these procedures.

Third, the analysis shows that anesthesiologists, surgeons, and nurses are the main contributors to this field. Practitioners should establish interdisciplinary teams to address postoperative sleep issues. For example, anesthesiologists can optimize anesthetic regimens to reduce circadian rhythm disruption, while nurses can implement nonpharmacological sleep interventions during inpatient care. In addition, the identification of emerging topics such as ERAS provides direction for future clinical research. Practitioners can design prospective studies to verify the effects of postoperative sleep within ERAS protocols.

However, this study inevitably has some limitations. It was impossible to enroll all publications in this field, and we restricted the paper type to research and review papers. Therefore, the results of this study represent the general situation of postoperative sleep research but cannot precisely reflect all details. We did not use processed parameters such as the Hirsch index to define the top authors and institutions because the primary function of bibliometric analysis is to provide a macrolevel view of a research field. Citation indicators may be influenced by the time of publication, meaning that recently published studies may have been underestimated. The number of citations increased dynamically over time. Thus, bibliometric analyses in this field must be updated in the future.

### Conclusions

This study presents the current research status and possible research directions through a visual bibliometric analysis of research on postoperative sleep over the past 10 years. There has been a large amount of literature on postoperative sleep over the past 10 years. Authors and organizations from China are leading contributors, followed by those from the United States. Anesthesiology is a critical discipline in this field. Postoperative pain is closely related to postoperative sleep and has become a hot topic. Recent studies have mainly focused on lung and breast cancer surgeries, and ERAS has become an emerging keyword.

## Abbreviations

ERAS: enhanced recovery after surgery
WoS: Web of Science
